# Circulating sclerostin is not suppressed following a single bout of exercise in young men

**DOI:** 10.14814/phy2.13695

**Published:** 2018-05-20

**Authors:** Katelyn I. Guerriere, Julie M. Hughes, Erin Gaffney‐Stomberg, Jeffery S. Staab, Ronald W. Matheny

**Affiliations:** ^1^ Military Performance Division United States Army Research Institute of Environmental Medicine Natick Massachusetts

**Keywords:** Bone formation, exercise, military, sclerostin

## Abstract

The aim of this study was to determine whether an acute bout of exercise reduces serum sclerostin under diet‐controlled conditions that stabilize the parathyroid hormone (PTH)‐1,alpha‐hydroxylase axis. Fourteen male volunteers (age, 22.1 years ± 4.05; BMI, 27.3 kg/m^2^ ± 3.8) completed a randomized crossover study in which they performed 10 sets of 10 repetitions of plyometric jumps at 40% of their estimated one‐repetition maximum leg press or a nonexercise control period. A calcium‐controlled diet (1000 mg/day) was implemented prior to, and throughout each study period. Blood was drawn for analysis of serum sclerostin, Dickkopf‐1, markers of bone metabolism (PTH, calcium), markers of bone formation (bone alkaline phosphatase, BAP; osteocalcin, OCN), and markers of bone resorption (tartrate‐resistant acid phosphatase 5b, TRAP5b; C‐telopeptide cross‐links of type I collagen, CTX) at baseline and 12, 24, 48, and 72 h following exercise or rest. Changes in serum concentrations were expressed as percentage change from individual baselines. Data were analyzed using a repeated measured linear mixed model to assess effects of time, physical activity status (rest or exercise condition), and the time by activity status interaction. There was a significant effect of exercise on OCN (*P* = 0.005) and a significant interaction effect for CTX (*P* = 0.001). There was no effect of exercise on any other biochemical marker of bone metabolism. A single bout of plyometric exercise did not induce demonstrable changes in biochemical markers of bone metabolism under conditions where dietary effects on PTH were controlled.

## Introduction

Sclerostin is a negative regulator of bone formation and a potent inhibitor of the anabolic Wnt signaling pathway (Bonewald and Johnson 2008; Robling et al. 2008). Sclerostin is primarily secreted by osteocytes – the resident cells in bone (Bonewald and Johnson 2008; Robling et al. 2008). The importance of Wnt signaling in bone homeostasis is demonstrated in cases of van Buchem's disease and sclerosteosis in which mutations in the *SOST* gene result in low basal levels of sclerostin and consequently, greatly enhanced bone formation (Weivoda and Oursler 2014). Sclerostin expression is regulated by several factors including estrogen, parathyroid hormone (PTH), and mechanical loading (Yu et al. 2011; Moustafa et al. 2012; Kim et al. 2015). Of these factors, mechanical loading may provide nonpharmacological means of suppressing sclerostin and thereby enhance bone anabolism (Zaman et al. 2012). In support of this notion, animal studies have consistently demonstrated that mechanical loading suppresses SOST expression and sclerostin production (Bonnet et al. 2009; Moustafa et al. 2012; Tu et al. 2012) and attenuates the loss of bone typically seen with disuse models such as hind limb unloading (Macias et al. 2012).

In humans, studies of chronic exercise indicate that long‐term mechanical loading may play a role in regulating sclerostin. In one study, 8 weeks of exercise training reduced serum sclerostin levels by over 33% in premenopausal women (Ardawi et al. 2012). Similarly, 12 months of resistance training twice a week, or 12 months of completion of a jump protocol three times a week, resulted in a 7% decrease in serum sclerostin in men (Hinton et al. 2017). Conversely, a study in postmenopausal women showed no change in sclerostin following a 12 month aerobic exercise intervention (Bergstrom et al. 2012). There are few studies regarding sclerostin responses to acute exercise, and results are variable; reporting elevation (Falk et al. 2016; Gombos et al. 2016; Pickering et al. 2017), no effect (Kerschan‐Schindl et al. 2015; Dekker et al. 2017), or suppression (Kerschan‐Schindl et al. 2015) following a single bout of loading. While the underlying cause of this variability in response is not known, it may be due, at least to some degree, to other mediators of sclerostin production such as estrogen (Modder et al. 2011; Kim et al. 2015) and PTH (Keller and Kneissel 2005; Yu et al. 2011).

PTH is a potent regulator of sclerostin expression in osteocytes (Yu et al. 2011) and is regulated in part by circulating concentrations of ionized calcium (Ca), which in turn, are sensitive to dietary intake of Ca (Brown 1991). Prior studies assessing the response of circulating sclerostin to an acute bout of exercise lack dietary control prior to and following exercise, which may impact circulating sclerostin through changes in PTH. Accordingly, we conducted a study of acute exercise with a controlled diet 3 days prior to and following exercise and rest conditions to minimize fluctuations in serum Ca and stabilize PTH with the aim of determining the influence of mechanical loading on acute changes in circulating sclerostin. We also investigated the influence of an acute bout of exercise on biochemical markers of bone formation and resorption. We hypothesized that an acute bout of exercise would reduce serum concentrations of sclerostin in the days following exercise when controlling dietary intake of Ca. Additionally, we hypothesized that an acute bout of exercise would increase concentrations of biochemical markers of bone formation and reduce serum concentrations of biochemical markers of bone resorption.

## Materials and Methods

The study was approved by the Institutional Review Board at the United States Army Institute of Environmental Medicine, and written informed consent was obtained for all volunteers prior to participation. The investigators have adhered to the policies for protection of human subjects as prescribed in Army Regulation 70‐25, and the research was conducted in adherence with the provisions of 32 CFR Part 219.

### Design

**Figure 1:**
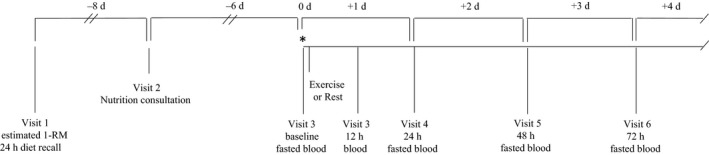
Timeline of study procedures. *Participants are randomly assigned to exercise or rest for visits 3–6. After a 1‐week period participants complete the alternate assignment of exercise or rest for visits 7–10 which mirror 3–6 on the timeline above.

To assess the effect of an acute bout of exercise on serum sclerostin and bone turnover marker concentrations, we performed a randomized crossover study in which participants completed two preliminary visits (visits 1 and 2) to collect background information, assess dietary intake, and complete an estimated one‐repetition maximum (1‐RM) test on a 3PQ, horizontal Plyo Press (Athletic Republic, Park City, UT). Participants were then assigned to complete a rest phase and an exercise phase, in random order. Each phase consisted of five visits over four consecutive days (visits 3–6 and 7–10) with a 1‐week period between phases (Fig. 1). Participants were asked to abstain from exercise and alcohol for 3 days prior to and for the duration of each testing phase for a total of seven consecutive days for each phase. Participants were also asked to abstain from taking nonsteroidal anti‐inflammatory drugs (NSAIDs) and nutritional supplements for the duration of their enrollment in the study (4–6 weeks).

### Participants

Fourteen healthy young men between the age of 18 and 39 were recruited to participate in the study. All participants were Active Duty Army Soldiers residing on an Army base. While they were not monitored continuously, daily activities, such as meal times and participation in physical activity, were largely regulated in alignment with study protocol. Participants were provided with instructions to limit physical activity outside of participation in study‐related exercise. Participants were free of endocrine, cardiopulmonary, and bone‐modifying diseases, and had self‐identified as not having used glucocorticoids in the 2 years prior to enrollment.

### Dietary control

Diet, specifically Ca intake, was controlled during the study using 24‐h recalls and 3‐day food records to estimate daily Ca intake preceding testing days, as well as by providing a Ca‐controlled diet to participants during testing days.

During visit 1, a registered dietitian met individually with each participant and performed a 24‐h recall to estimate the participant's typical daily Ca intake by identifying the number of servings of Ca‐rich foods/beverages the participant consumed. Participants took home a 2‐day food record that was reviewed at visit 2 for adherence to guidance provided by the dietitian. Participants subsequently filled out 3‐day food records prior to each testing phase. The food record data were entered into a food analysis software program, (Food Processor, ESHA Research, Salem, OR), and nutritional composition of the participants’ daily food intake was analyzed. For each participant, average daily nutrient intake was calculated from baseline records (visit 2) and prior to each testing period.

During the three full days of each testing period (visits 3–6 and visits 7–10), participants were provided a standardized diet using an individualized 3‐day cycle menu comprised of three commercially available meals per day and snacks. The standardized diets were developed by a registered dietitian and formulated to provide adequate energy for weight maintenance (30 kcal/kg), protein (0.8–1.0 g/kg), and Ca (1000 mg) per day. Menus for individual participants were identical between the two testing phases. All foods and beverages included in the menus were chemically analyzed for calorie, protein, vitamin D, and Ca content by an outside laboratory (Covance Inc., Princeton, NJ). Compliance with dietary regulations were assessed at each visit by a registered dietician.

### Bone biochemical markers

Fasted baseline blood samples were obtained between 0700 and 0800 for all visits (visits 3–6 and 7–10), and time of blood draw was kept consistent for each participant throughout all testing days. Participants were not fasted for the 12‐h blood draw on visit 3. Doing so would not allow for adequate time to consume provided meals. Each blood sample was drawn into 1, 10‐mL vacuum tube (Vacutainer, Becton Dickinson, Franklin Lakes, NJ, USA), allowed to clot at room temperature, centrifuged, and sera aliquots were stored at −80°C until analysis. Enzyme‐linked immunosorbent assays (ELISA) were performed using a Dynex DS‐2 immunoassay system for bone alkaline phosphatase (BAP, Quidel 8012), total osteocalcin (OCN, ALPCO 43‐OSNHU‐E01), tartrate‐resistant acid phosphatase (TRAP5b, Quidel 8033), C‐telopeptide cross‐links of type I collagen (CTX, Immunodiagnostic Systems AC‐02F1), Dickkpopf‐1 (DKK‐1, ALPCO DKK‐1), and sclerostin (R&D Systems SOST). Intact parathyroid hormone (PTH) was assayed on a Siemens Immulite 2000 system (L2KCO2). Serum calcium (Ca) and albumin were assayed on a Siemens Dimension Xpand Plus clinical chemistry system. Albumin‐adjusted calcium (ACa) was calculated using the formula, ACa = [(4−albumin)*0.8] + calcium. Interassay coefficients of variation (CV) were as follows for the ELISAs: BAP 3.09%, OCN 3.63%, TRAP5b 4.37%, CTX 7.45%, DKK‐1 5.68%, and sclerostin 2.90%. The CV for the PTH assay on the Immulite was 4.48%. The Dimension CV was 0.80% for Ca. All samples were run in duplicate or quadruplicate.

### Estimated 1‐RM

Participants completed an estimated 1‐RM (Thompson 2010) on visit 1 using a 3PQ Plyo Press (Athletic Republic, Park City, UT), which acts as a horizontal leg press with a suspension allowing participants to make a resisted maximal effort jump from a platform. Participants positioned themselves comfortably on the machine and test administrators made adjustments to the seat position so the tibia and femur were at approximately a 90° angle. Participants were brought through a standardized warm‐up before beginning the estimated 1‐RM protocol. The estimated 1‐RM protocol was completed consistent with American College of Sports Medicine Guidelines for Exercise Testing and Prescription 9th edition (Thompson 2010), where resistance was increased incrementally with consideration of difficulty of the previous set. Exercise was ceased when participants completed 3–5 repetitions at near‐maximal effort or failed to maintain proper form during the lift. The weight of the resistance and the repetitions completed were used to determine estimated 1‐RM (Thompson 2010).

### Plyo‐jump exercise protocol

Participants completed the exercise protocol on the first visit of the exercise phase, within 1 h of the baseline blood draw. Test administrators positioned the seat so that participants were in a starting knee flexion of approximately 90°. Participants were led through a 5–10 min warm‐up consisting of leg presses, lightly weighted Plyo Press jumps and stretching. Jump weight was calculated at 40% of estimated 1‐RM. Upon verbal command, participants commenced 10 sets of 10 repetitions of maximal effort jumps at 40% of their estimated 1‐RM. A timed 2 min rest was mandated between each set. This protocol was modeled after a similar exercise study that reported changes in markers of muscle metabolism following a single bout of plyometric training (Nindl et al. 2012).

### Statistical analyses

Statistical analyses were performed using SAS Version 9.4 software (SAS Institute Inc., Cary, NC). All biochemical markers were normalized to individual baseline values. A repeated measures linear mixed model was used to assess the effects of time, exercise, and their interaction. Within the model, time – as measured by visit number – was treated as a categorical variable with the first visit treated as a reference value and exercise was treated as a binary variable with no exercise used as the reference value. A *P*‐value <0.05 was considered significant. Data are presented as means ± standard deviations.

## Results

### Participants

Fourteen healthy young men completed the study (participant characteristics presented in Table 1). All participants reported being physically active in the 6 months prior to data collection. As reported in Table 1, average daily dietary intakes of total kcals, vitamin D, and Ca were not statistically difference between the rest and exercise phases.

**Table 1:** Participant characteristics

Characteristic	Mean (SD)
Age (years)	22.1 (4.05)
Height (cm)	174.6 (8.10)
Weight (kg)	83.0 (11.7)
BMI (kg/m^2^)	27.3 (3.8)
Race (*n*)	
Black	3
White	6
Other	5
Physical activity status (*n*)	
3–4 days/week	6
5+ days/week	8
Estimated 1‐RM (lbs)	529.1 (158.4)
Jump weight (40% 1‐RM, lbs)	208.9 (55.0)
Total dietary intake (rest, kcals)	2388 (616)
Total dietary intake (exercise, kcals)	2612 (640)
Dietary calcium intake (rest, mg)	1154 (317)
Dietary calcium intake (exercise, mg)	1223 (393)
Dietary vitamin D intake (rest, IUs)	285 (145)
Dietary vitamin D intake (exercise, IUs)	361 (165)

### Biochemical markers of bone metabolism

**Figure 2:**
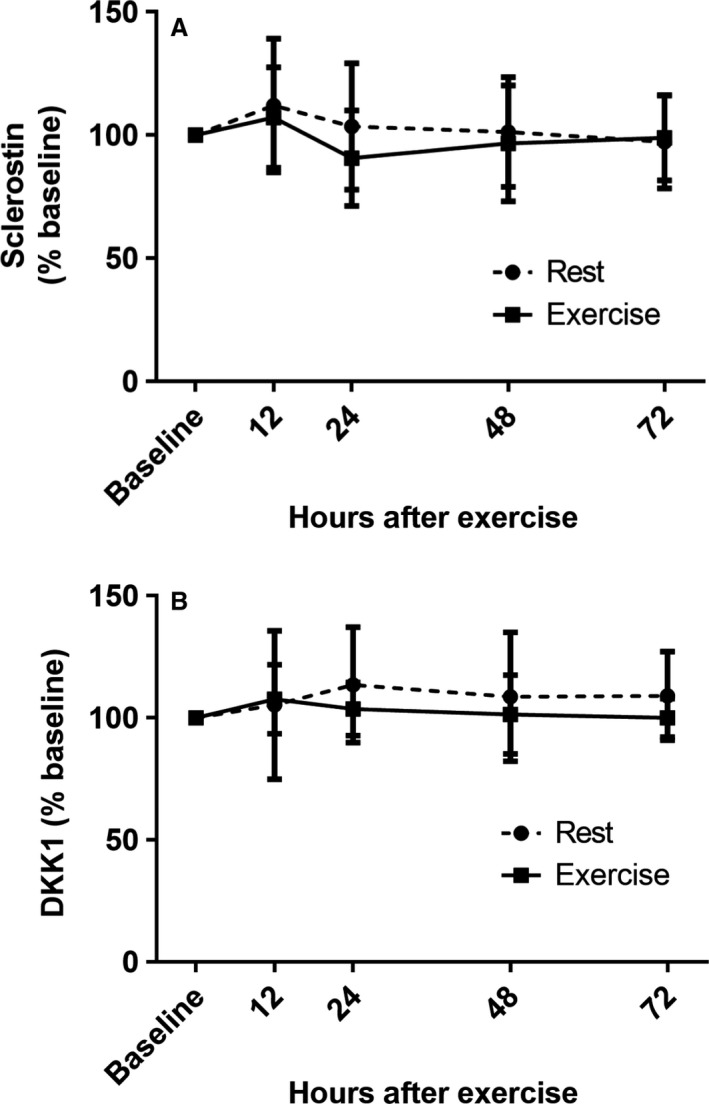
: Serum sclerostin (A) and DKK‐1 (B) expressed as a percent of baseline prior to exercise (baseline) and at 12, 24, 48, and 72 h postexercise. Values expressed as mean ± SD.

**Figure 3:**
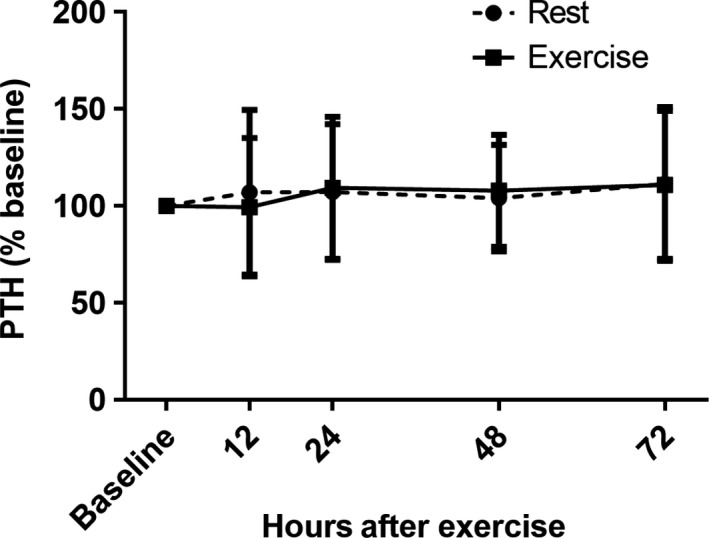
Serum values of intact PTH expressed as a percent of baseline prior to exercise (baseline) and at 12, 24, 48, and 72 h postexercise. Values expressed as mean ± SD.

**Figure 4:**
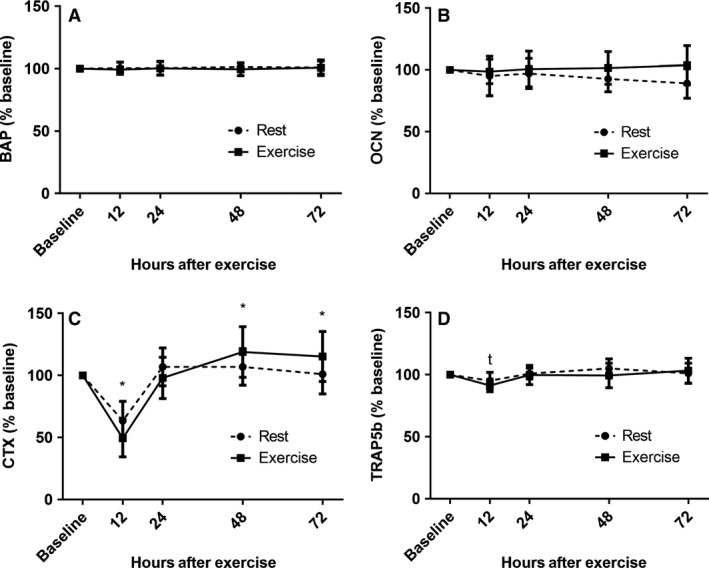
Serum values of biochemical markers of bone formation (BAP (A), OCN (B)) and resoprtion (CTX (C), TRAP5b (D)) expressed as a percent of baseline prior to exercise (baseline) and at 12, 24, 48, and 72 h postexercise. Values expressed as mean ± SD. * = interaction effect; *P* < 0.05, *t* = time effect; *P* < 0.0005.

We assessed two circulating biochemical markers known for their inhibition of the Wnt signaling pathway: serum sclerostin and DKK‐1. Figure 2 depicts serum sclerostin (A) and DKK‐1 (B) concentrations at 0, 12, 24, 48, and 72 h during both the rest and exercise conditions. There were no significant effects of time or exercise on sclerostin or DKK‐1 (*P* > 0.05 for all). Additionally, there were no significant effects of time or exercise for PTH (Fig. 3) or BAP (Fig. 4A) (*P* > 0.05 for all). However, there was a significant main effect of time for TRAP5b (*F* = 8.05, *P* < 0.0001), driven by a decrease in concentrations at 12 h (Fig. 4D). There was a significant main effect of exercise on OCN (*F* = 9.62, *P* = 0.005), however, there was no interaction of time by exercise at any time point (Fig. 4B). Lastly, there was a significant main effect of time for CTX (*F* = 62.82, *P* < 0.0001), as well as a time by exercise interaction effect (*F* = 4.80, *P* = 0.001) (Fig. 4C). Raw concentration data for all biochemical markers are provided in Table 2.

**Table 2:** Raw concentrations of biochemical markers

Biochemical marker	Condition	Time point, concentrations				
		Baseline	12 h	24 h	48 h	72 h
Sclerostin (pg/mL)	Rest	140.38 (47.22)	151.83 (48.46)	143.23 (57.58)	137.01 (39.52)	131.22 (32.70)
	Exercise	142.81 (49.70)	148.83 (45.61)	128.72 (50.54)	133.95 (40.20)	139.48 (49.87)
DKK‐1 (pmol/L)	Rest	63.43 (23.71)	62.81 (21.30)	69.66 (24.91)	66.94 (26.93)	67.74 (25.62)
	Exercise	61.07 (21.07)	65.54 (23.76)	62.77 (20.48)	61.03 (21.22)	60.72 (23.08)
OCN (ng/mL)	Rest	20.80 (8.75)	19.36 (8.03)	20.01 (8.67)	19.21 (8.42)	18.53 (8.42)
	Exercise	20.25 (8.53)	20.12 (9.18)	20.20 (8.64)	20.08 (7.74)	20.27 (7.10)
BAP (U/L)	Rest	29.38 (11.81)	29.47 (11.86)	29.31 (11.30)	29.84 (12.21)	29.74 (12.53)
	Exercise	30.40 (13.04)	30.06 (12.57)	30.00 (11.62)	29.86 (11.72)	30.25 (11.99)
CTX (ng/mL)	Rest	0.95 (0.39)	0.59 (0.26)*	1.01 (0.44)	1.02 (0.47)	0.96 (0.42)
	Exercise	0.94 (0.49)	0.48 (0.25)*	0.93 (0.49)	1.09 (0.49)	1.07 (0.51)
TRAP5b (U/L)	Rest	3.83 (1.80)	3.60 (1.58)	3.85 (1.73)	4.02 (1.87)	3.86 (1.74)
	Exercise	3.67 (1.73)	3.30 (1.47)	3.58 (1.51)	3.59 (1.60)	3.72 (1.62)
PTH (pg/mL)	Rest	25.43 (4.86)	27.04 (11.47)	27.00 (9.43)	25.95 (6.89)	28.15 (11.13)
	Exercise	26.96 (11.81)	23.89 (7.28)	26.31 (7.31)	26.79 (9.37)	27.54 (10.41)
Ca (mg/dL)	Rest	9.73 (0.22)	9.61 (0.29)	9.66 (0.23)	9.54 (0.27)	9.52 (0.21)
	Exercise	9.60 (0.29)	9.72 (0.35)	9.56 (0.25)	9.51 (0.28)	9.59 (0.25)
Alb (mg/dL)	Rest	4.09 (0.21)	4.11 (0.15)	4.08 (0.15)	4.07 (0.13)	4.03 (0.15)
	Exercise	4.11 (0.22)	4.15 (0.21)	4.07 (0.20)	4.07 (0.19)	4.11 (0.18)
ACa (mg/dL)	Rest	9.66 (0.25)	9.523 (0.29)	9.60 (0.24)	9.48 (0.25)	9.50 (0.19)
	Exercise	9.51 (0.24)	9.60 (0.33)	9.51 (0.24)	9.46 (0.26)	9.51 (0.26)

All data are presented as mean(SD).

DKK‐1, dickkopf‐1; OCN, total osteocalcin; BAP, bone specific alkaline phosphatase; CTX, c‐telopeptide cross‐links of type 1 collagen; TRAP5b, tartrate‐resistant acid phosphatase 5b; PTH, intact parathyroid hormone; Ca, total calcium; Alb, albumin; ACa, albumin‐adjusted calcium.

*Significantly different from baseline (*P* < 0.001).

## Discussion

The primary aim of this study was to investigate changes in serum sclerostin and biochemical markers of bone formation and resorption following an acute bout of exercise, independent of changes in serum PTH. To achieve this aim, we controlled dietary calcium intake and evaluated serum concentrations of sclerostin and other biochemical markers of bone turnover at several time points up to 72 h following rest and exercise. In contrast to our hypotheses, we observed no exercise‐induced changes in serum sclerostin or other biochemical markers of bone metabolism.

While we report no change in circulating sclerostin in young men after 100 horizontal, weighted jumps, other studies have reported mixed results, including elevation (Falk et al. 2016; Gombos et al. 2016; Pickering et al. 2017), suppression (Kerschan‐Schindl et al. 2015), or no change (Dekker et al. 2017) in circulating concentrations of sclerostin following an acute bout of exercise. This may be due, in part, to varying study designs. One significant variation in design among studies that evaluated changes in circulating sclerostin in response to acute exercise involves dietary regulation of Ca. Dietary intake of Ca influences serum concentrations of ionized Ca, which in turn influences serum concentrations of PTH (Brown 1991). PTH is a potent regulator of sclerostin (Keller and Kneissel 2005; Yu et al. 2011; Tamura and Kaji 2013), and therefore, dietary regulation of Ca may prevent fluctuations in sclerostin that do not reflect mechanical stimulation alone. For example, it is possible that in an ultramarathon, the dietary and metabolic demands of such a rigorous and extended physical activity results in declines in ionized Ca, increases in PTH, and decreased sclerostin concentrations independent of the physical activity. In our study, we regulated dietary intake of Ca and measured PTH at each time point, observing no changes in PTH, as intended. Therefore, our results likely reflect the effects of the mechanical stimulus alone.

Another difference across studies is the timing of blood collection following exercise. For example, several studies reported a range of increases in sclerostin immediately postexercise (within 5 min of exercise cessation) of ~8–50% (Falk et al. 2016; Gombos et al. 2016; Pickering et al. 2017), including a study with a similar design of 144 plyometric jumps (Falk et al. 2016). In that study, serum sclerostin concentrations increased by 50%, 5 min postexercise but returned to baseline levels at 1 h postexercise and remained at baseline values 24 h postexercise (Falk et al. 2016). We did not assess changes in circulating sclerostin immediately post exercise and therefore may have missed a narrow window of change following a single bout of exercise.

Another possible explanation for discrepancies in sclerostin responses to acute exercise is the varying characteristics of the exercise stimulus. In studies that have reported changes in sclerostin following acute exercise, modalities have included a 45‐min treadmill run (Pickering et al. 2017), 46 min of resistance exercise or brisk walking (Gombos et al. 2016), 144 plyometric jumps (Falk et al. 2016), and a 34 h, 246 km ultramarathon race (Kerschan‐Schindl et al. 2015). All these studies reported increases in sclerostin immediately postexercise except for the study in ultramarathon runners which reported a 15% reduction in sclerostin concentrations 36 h after completion of the race (Kerschan‐Schindl et al. 2015). Therefore, it is possible that the overall duration and intensity of our exercise protocol was not significant enough of a stimulus to result in appreciable changes in circulating sclerostin.

Like sclerostin, we also observed no effects of exercise on any biochemical marker of bone formation or resorption. The reported effects of acute exercise on circulating biochemical markers of bone formation and resorption are equivocal (Guillemant et al. 2004; Maimoun et al. 2006; Scott et al. 2010, 2011, 2013; Kish et al. 2015; Sale et al. 2015), and we speculate that this may be at least partially explained by differences in study designs. We observed an overall effect of exercise on OCN as well as an overall effect of time on TRAP5b and an interaction effect on CTX. As expected, CTX was influenced by diurnal changes. The interaction effect could signify an increase in bone resorption with exercise at 48 and 72 h postexercise. Similar findings have been reported in other studies (Scott et al. 2010; Kohrt et al. 2018); however, TRAP5b, another marker of bone resorption did not change appreciably with exercise. The significance of the effect of time on TRAP5b remains unclear; however, it is possible that this effect was driven by the 12‐h time point, at which TRAP5b was significantly reduced in both control and exercise conditions. We also observed an effect of exercise on serum OCN, with an approximately 9% decrease from baseline under the rest condition as opposed to a relatively stable response under the exercise condition. It is possible that the decrease seen under the rest condition is an effect of the sedentary behavior of the participants for the 6 days prior to the 72 h blood draw and that the single bout of exercise was enough to mitigate that decline under the exercise condition.

The strengths of this study include the crossover design with random assignment to the order of rest and exercise in a sample of male participants. Additionally, this study closely regulated dietary intake of Ca with the aim of stabilizing PTH in order to isolate the effects of mechanical loading on serum sclerostin. One limitation of this study, however, is that we did not make use of an uncontrolled, ad libitum condition for comparison. An uncontrolled condition would have allowed for evaluation of the potential role of PTH in the response of sclerostin to exercise. Another limitation is that we did not measure ionized Ca which plays an important role in regulating PTH (Kohrt et al. 2018). Nevertheless, ionized Ca was likely stable throughout the study given the stability in total Ca and PTH. Another potential limitation is that we did not directly calculate plasma volume changes that may have occurred with exercise as observed in prior studies (Brahm et al. 1997; Sherk et al. 2017). However, as we did not observe any significant changes in serum albumin, which tends to inversely change with changes in plasma volume (Brahm et al. 1997), we can infer that there was likely little plasma volume change at the sampling time points. Finally, we evaluated only circulating sclerostin (and markers of bone resorption and formation) and not sclerostin at the cell or tissue level. Circulating sclerostin concentrations are predicated on a number of regulatory processes (including transcription, protein synthesis, secretion, and turnover), and alteration(s) in one or several of these processes may have occurred.

In summary, we showed no significant response in serum concentrations of sclerostin and biochemical markers of bone formation and resorption related to an acute bout of plyometric jump exercise. These findings suggest that the single bout of plyometric exercise performed in this study may not have been of long enough duration or potent enough of a stimulus to appreciably alter serum concentrations of sclerostin in young, healthy men.

## Conflict of Interest

All authors have nothing to disclose. The views expressed in this manuscript are those of the authors and do not reflect the official policy of the Department of Army, Department of Defense, or the U.S. Government.
